# L’Atrésie colique: à propos de deux cas

**Published:** 2010-11-04

**Authors:** Karima Atarraf, Abdelkarim Shimi, Maryem Lachqar, Mustapha Harandou, Youssef Bouabdallah

**Affiliations:** 1 Service de Chirurgie Pédiatrique, CHU Hassan II, Fès, Maroc; 2 Service d’Anesthésie Réanimation, CHU Hassan II, Fès, Maroc

**Keywords:** Atrésie, colon, Maroc, chirurgie, appareil digestif

## Abstract

L’atrésie colique est la moins fréquente des atrésies de l’appareil digestif. Le trouble de vascularisation anténatal est l’hypothèse étiologique la plus communément admise pour expliquer les formes complètes. Cette anomalie peut être isolée ou faire partie d’un syndrome poly malformatif. Nous rapportons deux cas d’atrésie du colon: le premier admis dans un tableau d’occlusion néonatale basse, épreuve à la sonde négative. L’exploration chirurgicale a objectivé une atrésie colique type I. le patient a bénéficié d’une colostomie première puis d’une anastomose termino-terminale. Avec des suites opératoires simples. Le deuxième patient a été admis dans un tableau d’occlusion néonatale haute. L’atrésie colique était de découverte fortuite en per-opératoire, associée à une atrésie grêlique. Le patient a bénéficié d’une double stomie, mais les suites opératoires ont été marquées par le décès dans un tableau de choc septique. A la lumière des ces deux observations et après une revue de la littérature; Nous soulignons la rareté de la pathologie et la difficulté de prise en charge surtout en cas de retard diagnostic.

## Introduction

L’atrésie colique est une malformation congénitale rare. Elle se définit par l’absence congénitale de lumière dans le côlon. Elle ne représente que 1,8 à 15% de l’ensemble des atrésies intestinales [1,2]. Son association à une atrésie du grêle est exceptionnelle.

## Observations

### Observation 1

**Figure 1:**
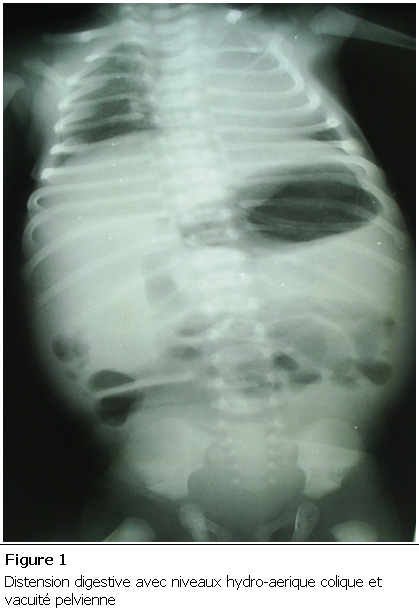
Distension digestive avec niveaux hydro-aériques coliques et vacuité pelvienne

**Figure 2:**
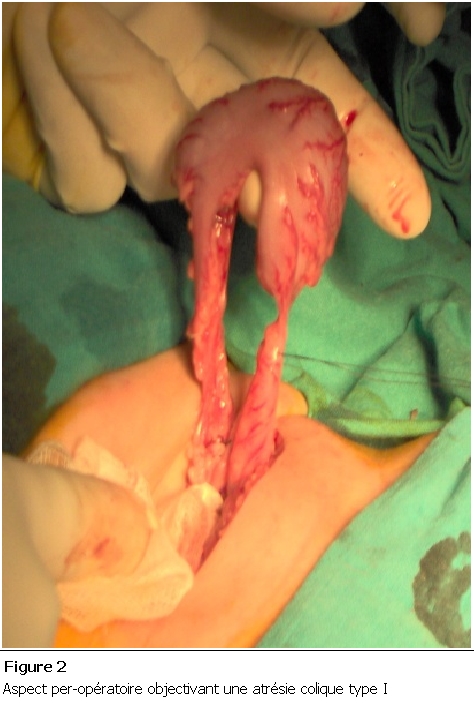
Aspect per-opératoire objectivant une atrésie colique type I

**Figure 3:**
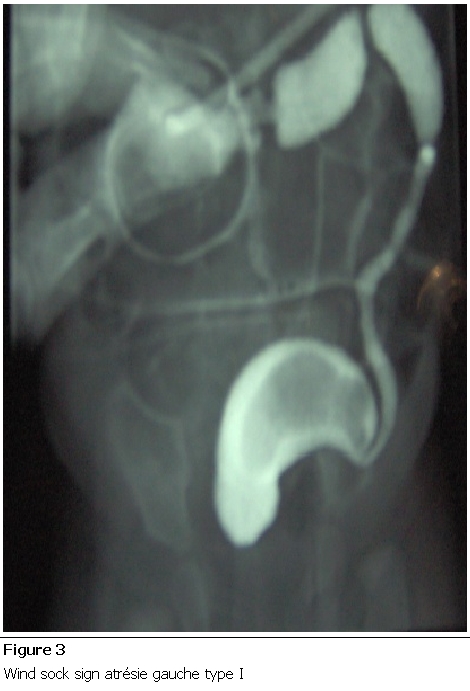
Wind sock sign atrésie gauche type I

**Figure 4:**
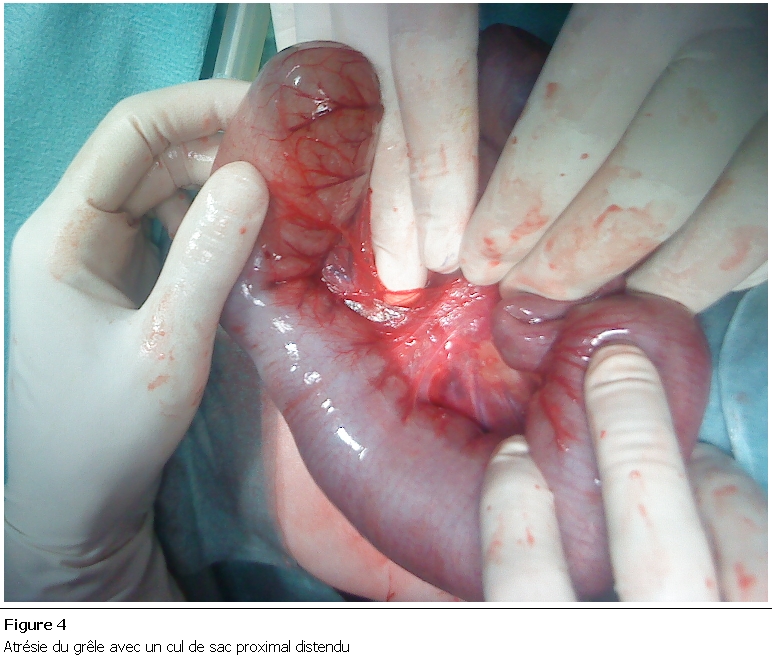
Atrésie du grêle avec un cul de sac proximal distendu

Nouveau-né de sexe féminin, grossesse non suivie se disant à terme. Admis à J5 de vie dans un tableau d’occlusion néonatale basse à ventre ballonné, avec notion d’absence d’émission de méconium et une épreuve à la sonde négative. La radiographie thoraco-abdominale montrait des niveaux hydro-aériques type colique (Figure 1). L’exploration chirurgicale a trouvé un diaphragme au niveau du côlon descendant (Figure 2), une colostomie droite a été réalisée. Ultérieurement une opacification par voie basse a été faite, ayant objectivé une atrésie du côlon gauche (Figure 3). La patiente a bénéficié dans un second temps d’une résection du côlon atrétiQue avec anastomose termino-terminale (Figure 4), et d’un rétablissement de continuité. Les suites postopératoires étaient simples avec un recul de 2ans.

### Observation 2

**Figure 5:**
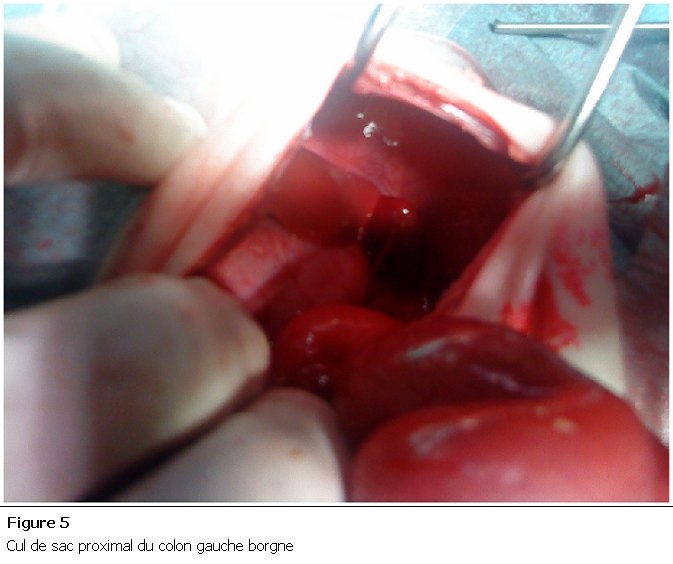
Cul de sac proximal du colon gauche borgne

**Figure 6:**
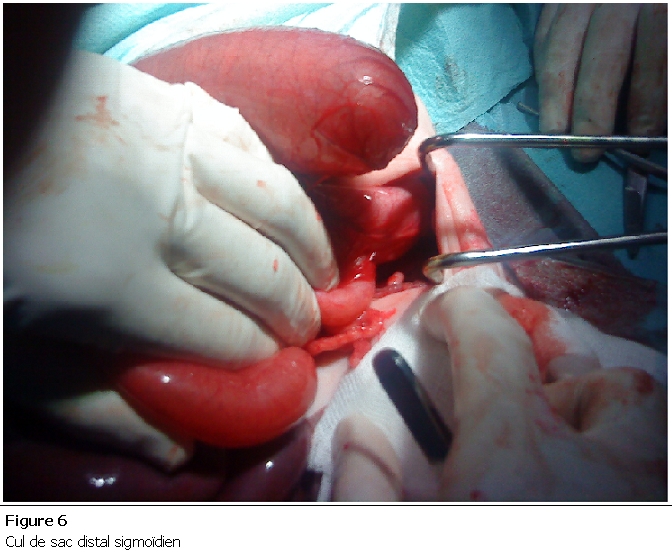
Cul de sac distal sigmoïen

Nouveau né de sexe masculin, admis à J3 de vie pour prise en charge d’une infection néonatale avec par ailleurs des vomissements bilieux, une absence d’émission du méconium et un abdomen plat à l’examen clinique. La radiographie thoraco-abdominale face debout montrait deux niveaux hydro-aériques grêlique avec absence d’aération digestive ailleurs. Le bilan infectieux était positif (Protéine C Réactive à 123, Hyperleucocytose à 18000/mm). Le diagnostic d’une occlusion néonatale haute a été retenu et l’exploration chirurgicale a objectivé de multiples atrésies grêliques à partir de la 3é anse iléale (Figure 5) avec présence d’une atrésie colique à la jonction colon descendant et sigmoïde de type III (Figure 6). Une double stomie grêlique et colique a été réalisée chez lui. Le décès est survenu à J3 du postopératoire dans un tableau de choc septique.

## Discussion

Les atrésies coliques sont des malformations congénitales rares, elles sont définies par l’absence congénitale, complète ou non, d’un segment colique. Leur incidence est en moyenne de 1 à 2 cas par an. Etensel a noté une légère prédominance masculine avec un sexe-ratio de trois filles pour quatre garçons et un taux de prématurité estimé à 32 % [3,4]. Dans les autres séries, la plupart des nouveau-nés ont été rapportés être à terme [5], comme étant le cas de nos malades.

On distingue trois types d’atrésie colique: Type I: diaphragme muqueux, Type II: atrésie complète, le Type III: défet mésentérique en V, et Les atrésies multiples qui sont dites type IV [6]. Le type III est souvent localisé en amont de l’angle colique gauche, alors que les atrésies coliques en aval sont de type I ou II. L’étio-pathogénie est en fait complexe et probablement multifactorielle [7,8]. Plusieurs théories sont avancées mais le trouble de vascularisation anténatal est l’hypothèse étiologique la plus communément admise.

Un mécanisme compressif a été rapporté dans la littérature, qui fait état de deux observations d’atrésie du côlon transverse secondaire à une compression du mésentère par un kyste du cholédoque [9]. Une origine malformative a été aussi avancée pour expliquer les atrésies multiples [10]. Enfin certains auteurs ont évoqué une origine génétique concernant la non-expression du Ff10 (fibroblast growth factor) ou de son récepteur (Fgfr) [11].

Les malformations associées sont peu communes Type oculaires, cardiaques, paroi abdominale mais deux sont fréquentes : le Mégacôlon congénital et l’atrésie jéjuno-iléale, étant le cas de notre malade dont le tableau clinique était celui d’une occlusion néonatale haute.

Le diagnostic anténatal est difficile, basé essentiellement sur l’échographie objectivant, des signes indirects tels qu’un hydramnios par défaut d’absorption du liquide amniotique et une dilatation du segment digestif en amont de l’atrésie [7]. L’IRM foetale n’est pratiquée qu’en seconde intention en cas de doute échographique. Elle permet, en plus de la mensuration du diamètre intestinal, une meilleure analyse morphologique de l’intestin ainsi que de son signal. A l’heure actuelle, aucune description IRM de l’atrésie colique n’a été rapportée dans la littérature. En pratique, l’IRM est réalisée entre 23 et 38 SA, elle montre une dilatation des anses d’amont, dont le signal varie selon le siège de l’obstacle : liquidien dans les atrésies proximales et méconial si obstacle distal [12]. L’IRM contrairement à l’échographie, précise le niveau de l’atrésie et différencie entre atrésie colique et atrésie iléale distale, iléus méconial, maladie de Hirschprung et immaturité fonctionnelle du côlon.

Après la naissance le tableau clinique est celui d’une occlusion néonatale basse avec épreuve à la sonde négative. La radiographie thoracoabdominale permet d’objectiver des signes radiologiques d’une occlusion à type de niveaux hydroaériques et de distension grêlique. L’index baryté reste l’examen de référence permet un diagnostic topographique précis. (Siège et type).

Le traitement comprend deux phases et dépend surtout de l’état général du patient et de la précocité de prise en charge qui conditionne certaines complications : telle une perforation digestive, des troubles métaboliques ou une septicémie.

## Conclusion

L’atrésie colique est une urgence néonatale. Son évolution est généralement favorable dans les cas traités précocement et en dehors des malformations associées. Le diagnostic anténatal dans notre contexte n’est toujours pas de mise; d’où l’intérêt de l’opacification précoce devant toute occlusion néonatale basse avec épreuve à la sonde négative; seul garant du diagnostic et donc d’un traitement précoce.

## Conflits d’intérêts

Les auteurs déclarent n’avoir aucuns conflits d’intérêts.

## Contribution des auteurs

Karima Atarraf et Meryem lachqar ont participé à la prise en charge des deux malades , la prise des photos et la rédaction de l’article, Shimi Abdelkarim a participé à la recherche bibliographique et a la rédaction de l’article, Harandou mustapha et Youssef Bouabdallah ont participé à la prise en charge des deux malades.
